# Prostate cancer disparities in Black men of African descent: a comparative literature review of prostate cancer burden among Black men in the United States, Caribbean, United Kingdom, and West Africa

**DOI:** 10.1186/1750-9378-4-S1-S2

**Published:** 2009-02-10

**Authors:** Folakemi T Odedina, Titilola O Akinremi, Frank Chinegwundoh, Robin Roberts, Daohai Yu, R Renee Reams, Matthew L Freedman, Brian Rivers, B Lee Green, Nagi Kumar

**Affiliations:** 1 H. Lee Moffitt Cancer Center, Tampa, Florida, USA; 2 Federal Medical Center (FMC), Abeokuta, Nigeria; 3 Barts and The London NHS Trust and Newham University NHS Trust, London UK; 4 Princess Margaret Hospital in Nassau, The Bahamas; 5 Florida A&M University, Tallahassee, Florida, USA; 6 Dana-Farber Cancer Institute, Boston, Massachusetts, USA

## Abstract

**Background:**

African American men have the highest prostate cancer morbidity and mortality rates than any other racial or ethnic group in the US. Although the overall incidence of and mortality from prostate cancer has been declining in White men since 1991, the decline in African American men lags behind White men. Of particular concern is the growing literature on the disproportionate burden of prostate cancer among other Black men of West African ancestry in the Caribbean Islands, United Kingdom and West Africa. This higher incidence of prostate cancer observed in populations of African descent may be attributed to the fact that these populations share ancestral genetic factors. To better understand the burden of prostate cancer among men of West African Ancestry, we conducted a review of the literature on prostate cancer incidence, prevalence, and mortality in the countries connected by the Transatlantic Slave Trade.

**Results:**

Several published studies indicate high prostate cancer burden in Nigeria and Ghana. There was no published literature for the countries Benin, Gambia and Senegal that met our review criteria. Prostate cancer morbidity and/or mortality data from the Caribbean Islands and the United Kingdom also provided comparable or worse prostate cancer burden to that of US Blacks.

**Conclusion:**

The growing literature on the disproportionate burden of prostate cancer among other Black men of West African ancestry follows the path of the Transatlantic Slave Trade. To better understand and address the global prostate cancer disparities seen in Black men of West African ancestry, future studies should explore the genetic and environmental risk factors for prostate cancer among this group.

## Introduction

In the United States (US), an estimated 30,870 cases of prostate cancer are expected to occur among African American men in 2007, accounting for 37% of all cancers diagnosed in African American men [1]. Between 2000 and 2003, the average annual prostate cancer rate was 60% higher in African American men compared to White men [1]. In addition, African American men have the highest mortality rate compared to any other racial or ethnic group in the US, and 2.4 times higher than in White men. Although prostate cancer incidence and mortality rates have been declining in both African American and White men since 1991, possibly due to improved diagnostic techniques, better screening and improved surgical and radiologic treatments, the rates remain comparably higher among African American men. *Why does this disparity continue to exist among African American men in spite of the significant research to eliminate this disparity?*

The population-based studies on prostate cancer disparity have traditionally studied African American men only or compared African American men to other racial groups within the US. Although these studies have provided vital insights necessary to understanding the primary differences among the ethnic groups in the US, we are left with more questions than answers. With the problem of prostate cancer disparity persisting, it is crucial that we begin to use new paradigms to examine the problem of prostate cancer disparity from new perspectives. The prospect for a new paradigm/new perspectives emerge with the growing literature on the disproportionate burden of prostate cancer among other Black men (in other parts of the world, other than the US) who are related to African American men through the Transatlantic Slave Trade (TAS) [2-7], especially Black men of West African ancestry. Based on the World Health Organization (WHO)'s worldwide cancer data, West African men have much lower prostate cancer incidence and mortality compared to African American men. For example, compared to Nigerian men, African-American men are >10 times likely to develop prostate cancer and 3.5 times likely to die from the disease [8]. On the other hand, Silverberg and Lubera [9] reported high rates of prostate cancer mortality in Martinique, Trinidad & Tobago; while Glover *et al. *[10]reported high rates of prostate cancer incidence in the predominantly Afro-Caribbean population of Jamaica. Thus the variability in risk and mortality across these populations of West African descent suggests a potential and important influence of environmental/lifestyle factors acting on prostate cancer risk in these already susceptible populations. *Can this evolving international data hold the key to our better understanding of the etiology of unequal burden of this disease in African American men?*

### The Transatlantic Slave Trade (TAS) Relations

Based on the TAS, about 15 million people were transported as slaves from Africa, not counting those who died en-route to the Americas, Caribbean and Europe [7]. The TAS comprised the following three journeys: (1) Outward passage from Europe to Africa carrying manufactured goods; (2) Middle passage from Africa to the Americas/Caribbean carrying African captives and other commodities; and (3) Homeward passage carrying sugar, tobacco, rum, rice, cotton and other goods back to Europe. These slave routes included diverse countries. The European countries involved in the TAS were Portugal, France, Netherlands, Spain, Denmark, Norway and United Kingdom. Slaves were primarily imported from the African countries – Benin, Nigeria, Ghana, Gambia, Senegal, Mozambique, and Angola. The middle passage took the slaves from Africa to the American and Caribbean countries – Barbados, Cuba, Haiti, Dominican Republic, Netherlands Antilles, Trinidad and Tobago, Jamaica, Brazil, and the US. Most of the slaves ended up in South America or the Caribbean with about 500,000 transported to North America. Unfortunately, most of the slaves in the Caribbean, Central America and South America died while the slave population in North America had higher life expectancy [7].

### Objective

In this manuscript, we explored the burden of prostate cancer among the TAS population targeting Black men of West African Ancestry to answer the question:*Does the prostate cancer disparities seen in Black men around the world follow the path of the TAS*?

## Methods

To achieve the objective for this study, we conducted a literature review, summarizing the body of evidence on prostate cancer morbidity and mortality in countries with high population of Black men of West African ancestry, excluding the US. Our goal was not to provide a meta-analysis evaluation nor conduct a comprehensive review/critique of these studies. Our aim was to merely examine the available proof in an effort to better understand the burden of prostate cancer among Black men of West African ancestry. The study countries for our review were West African TAS populations (Benin, Nigeria, Ghana, Gambia, and Senegal), the United Kingdom and Caribbean Islands (Barbados, Cuba, Haiti, Dominican Republic, Netherlands Antilles, Trinidad and Tobago, Jamaica, and Brazil). A systematic search of the computerized database, MEDLINE, was conducted for each country from the originating date of MEDLINE to July 2008 using the following keywords: Black men, prostate cancer, prostate cancer risk, prostate cancer incidence; prostate cancer morbidity; and name of TAS country. The inclusion criteria were:

• Original studies and review articles;

• Publication in English;

• Relevance to prostate cancer risk, incidence, prevalence or mortality; and

• Sufficient quality and quantity of evidence based on study design and validity, for example, well designed randomized/non-randomized study with good evidence to support study conclusion.

Studies employing the World Health Organization (WHO) GLOBOCAN program of the International Agency for Research in Cancer (IARC) [11] were excluded from the review because the data from developing countries are unreliable. As noted in the GLOBOCAN 2002 report [11], several developing countries do not have available data for cancer incidence and mortality. Thus, data from neighboring countries in the same regions were used to estimate developing countries. For example, the incidence data for each West African country were estimated from the proportions of available data from the respective country as well as un-weighted averages of the observed rates (by sex and age-group) in registries from other West African countries. The cancer mortality for these countries was calculated as the product of incidence and the probability of dying from the disease. The pooled estimates of survival for the West African regions were applied to countries in these regions. Given the lack of reliable and valid cancer incidence and mortality data for most of the developing countries, the prostate cancer rates reported in the Globocan 2002 report [11] for these countries are underestimated.

The steps for the literature review were in the following order: Step 1 Search for relevant articles using specified keywords on MEDLINE; Step 2 Screening of Titles to eliminate irrelevant articles; Step 3 Screening of abstracts of all articles identified in Step 2 for relevance; and Step 4 Review of papers identified in Step 3 based on inclusion criteria by study investigators. Only the studies with study design ranked *Excellent*, *Good *or *Satisfactory *by authors were included in the review. Subsequently, the studies were grouped together and summarized according to TAS country. It is important to note that some of the studies included more than one (1) TAS country.

## Results

Results are presented for each region below.

### Tracing the root of prostate cancer disparities to West Africa

The lack of population-based cancer registries in developing countries, especially African countries, leaves only hospital-based prostate cancer morbidity and mortality data. Thus, most of the articles identified in our review presented only hospital-based data. There was no published study on prostate cancer morbidity and mortality in *Benin, Gambia*, and *Senegal*. Although there was no published study reporting prostate cancer morbidity and mortality in *Senegal*, Gueye *et al. *[12] found the clinical characteristics of prostate cancer among the Senegalese (median PSA of 37.0 ng/mL) to be different from American Blacks (median PSA of 6.3 ng/mL) and Whites (median PSA of 6.1 ng/mL); p < 0.001. Senegalese men had worse median (and mean) PSA level and worse tumor stage. As noted by the authors, these differences can possibly be explained by the considerable differences in early detection between US men and Senegalese men.

Reports on prostate cancer patterns in *Ghanaian *men were found in only two published studies. The first study was a review of the genitourinary cancers seen at the Accra Korle-Bu Teaching Hospital, Klufio [13]. The authors found prostate cancer to comprise about 64% (349 cases) of all genitourinary cancers from 1980 to 1990. In a more recent study, Wiredu and Armah [14] conducted a 10-year review (1991–2000) of all cancer mortality patterns at the same institution based on autopsy and death certification data, and found prostate cancer to be the second leading cause of cancer deaths (286 cases). None of the studies presented the actual incidence, prevalence or mortality from prostate cancer.

Contrary to the WHO IARC global ranking, several published studies indicate higher incidence of prostate cancer in *Nigerian *men. A total of 17 original papers, which met our review criteria, were found to document prostate cancer patterns in Nigeria. Odedina, Ogunbiyi and Ukoli previously reviewed all the 17 articles in 2006 [8]. The first published paper reviewed by Odedina, Ogunbiyi and Ukoli [8] was as early as 1981 and the latest study in 2005. The studies were conducted in different regions of Nigeria, including the North, Southeast, West, and in rural South. The authors concluded that the body of evidence from the 17 studies indicates high prostate cancer risk among Nigerian men and a prostate cancer burden similar to that of Black men in the US. Table 1 provides a summary of the prostate cancer incidence and prevalence rates reported in the review article.

**Table 1:** Comparison data on incidence and prevalence of prostate cancer in Black Men of West African ancestry

**Country**	**Author**	**Incidence rate per 100,00**	**Prevalence rate**	**Source of data**
**West Africa**				

Nigeria	Osegbe, 1998 [32]	**127.0**	-	Hospital-based data; for men 45 years or older

Nigeria	Ekwere & Egbe, 2002 [37]	**61.3**	-	Hospital-based data; for men 35 years or older

Nigeria	Eke & Sapira, 2002 [33]	**114.0**	-	Hospital-based data; for men 45 years or older

Nigeria	Ukoli et al., 2003 [26]	-	**15.7%**	Rural Nigerian population-based; PSA = 4 ng/mL for men 50 years & older

**Caribbean**				

Jamaica	Glover et al., 1998 [10]	**304.0**	-	Large population-based study; age-adjusted rate

Jamaica	Hanchard et al., 2001 [19]	**56.4**	-	Cancer registry data; age-adjusted rate

Tobago	Bunker et al., 2002 [22]	-	**10.0%**	Large population-based study; biopsy confirmed for men 40–79 years

Tobago	Bunker et al., 2004 [21]	-	**10.7%**	Large population-based study; biopsy confirmed for men 40–79 years

Brazil	Barros et al., 2003 [16]	-	**19.7%**	Hospital-based data; for men 40–79 years

**Europe**				

United Kingdom	Chinegwundoh et al., 2006 [24]	**647.0**	-	Population-based audit for African-Caribbean (all blacks); age-adjusted rate for men 50 years or older over 2 years

United Kingdom	Ben-Shlomo et al., 2007 [25]	**165.5**	-	Population-based audit for African-Caribbean (all Blacks); age-adjusted rate for men 50 years or older over 5 years

United Kingdom	Ben-Shlomo et al., 2007 [25]	**173.1**	-	Population-based audit for Black Caribbean; age-adjusted rate for men 40 years or older over 5 years

United Kingdom	Ben-Shlomo et al., 2007 [25]	**139.3**	-	Population-based audit for Black African (all blacks); age-adjusted rate for men 40 years or older over 5 years

**United States**				

US Black Men	ACS, 2008 [1]	**258.30**		SEER 2000 – 2003 data

### Tracing the root of prostate cancer disparities to Caribbean Islands

There were no published articles that met our review criteria for the countries *Barbados, Cuban, Dominican Republic, Haiti*, and *Netherlands Antilles*. Three published studies met the criteria for prostate cancer patterns in *Brazil*. The earliest study, in 1991, reviewed the Cancer Registry data of São Paulo between 1969 and1974 [15] and found Mulatto and Black men to be disproportionately affected by prostate cancer compared to White men. The authors concluded that the prostate cancer disparities seen among Mulattos and Blacks in Brazil were similar to the experiences of Blacks in the US. In another study of Blacks, Mulattos and Whites, Barros *et al. *[16] explored the prevalence of prostate cancer from 1999 to 2001 at the Federal University of Bahia's hospital. Based on a sample of 162 patients who had prostate biopsy, the authors found no statistically significant differences among the three ethnic groups on mean PSA level (22.0 ng/dl for White, 18.1 ng/dl for Mulatto, and 19.3 ng/dl for Black; p value of 0.65) and prostate adenocarcinoma prevalence (53.3% for White, 53.3% for Mulatto, and 64.9% for Black; p value of 0.36). The final study in Brazil targeted a population with a high number of Bantu African ancestors in São Paulo. Screening 473 volunteers between the age of 40 and 79, Paschoalin et al. [17] found the prevalence of prostate cancer to be greater among Blacks and Mulattos compared to Whites. The proportion of biopsy-confirmed prostate cancer among 121 patients were 8.5% Black men, 6.7% for Mulatto men, and 0.6% for White men (p = 0.006).

The classic study by Glover *et al. *[10] in 1998 provided a significant update on prostate cancer in *Jamaica*. Prior to this study, African American men were cited as having the highest rate for prostate cancer in the world. Glover *et al. *[10] conducted a 5-year retrospective review of over 1,000 prostate cancer cases between 1989 and 1994 in Kingston, Jamaica. The authors reported a mean-age adjusted incidence rate of 304 per 100,000 based on 1,121 prostate cancer cases retrieved from the Jamaican Cancer Registry, government pathology laboratory, hospital and clinic records, and physician office records. The authors concluded that the prostate cancer incidence in Kingston was significantly higher than that of the US Blacks. Glover *et al. *[10] confirmed the results from an earlier study by Brooks and Wolf [18], who found prostate cancer to be the most prevalent cancer among Jamaican men in Kingston and St. Andrew based on a review of all cancer cases from 1958 to 1987. More recently, Hanchard *et al. *[19] in 2001 reported prostate cancer as the leading site for incidence of cancer in males based on the 1941 malignant neoplasms recorded in Kingston and St. Andrew among males. The authors reported 619 cases of prostate cancer and age-standardized incidence rate of 56.4. In 2002, Blake *et al. *[20] also reported that prostate cancer continued to be the leading cancer mortality site among Jamaican men.

The three published studies found to be relevant for Trinidad and Tobago were by Bunker *et al. *[21-23]. In 2004, Bunker at al [21] reported a population-based prostate cancer study, screening on about half the population of Tobago men. Over 2,500 men between the age of 40 and 79 years were screened using serum prostate-specific antigen (PSA) and digital rectal examination (DRE). The authors found biopsy-confirmed prostate cancer prevalence in this population to be about 11% (277 out of 2,583 men). These results confirmed the findings of an earlier report on the same population by Bunker *et al. *[22] based on the screening of 2,484 men between the age of 40 and 79 in Tobago. In another report, Bunker *et al. *[23] compared the Tobago population of West African descent to the men of Asian-Indian descent in Trinidad, targeting men between 50 and 64. The prostate cancer prevalence based on population-based screening was found to be significantly higher for the Afro-Tobagonian men (8.3%) compared to the Asian-Indian men (2.3%). This difference was also observed on all age categories: 50–54; 55–59; and 60–64.

### Tracing the root of prostate cancer disparities to United Kingdom

**Figure 1:**
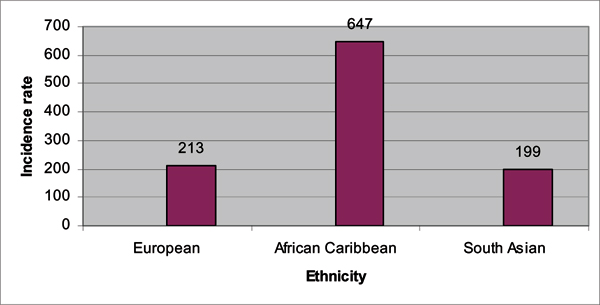
**Age-adjusted prostate cancer incidence rates comparing UK European, African Caribbean, and South Asian men, age 50 and above, between 1999 and 2000**. *Incidence rate is per 100,000. Source: Chinegwundoh F, Enver M, Lee A, Nargund V, Oliver T, Ben-Shlomo Y. Risk and presenting features of prostate cancer amongst African-Caribbean, South Asian and European men in North-east London. *BJU International *2006, **98**:1216–1220. [24]

**Figure 2:**
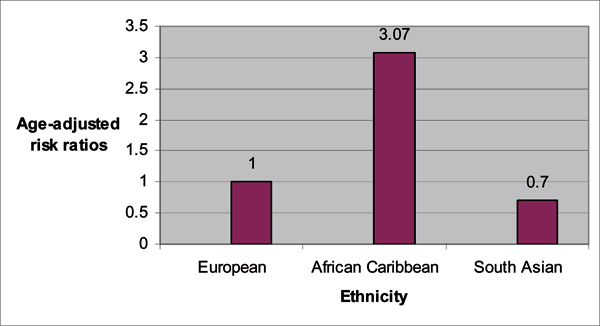
**Age-adjusted risk ratios for prostate cancer comparing UK European, African Caribbean, and South Asian men, age 50 and above, between 1999 and 2000**. *P value < 0.001 for European vs. African Caribbean. †P value = 0.13 for European vs. South Asian. Source: Chinegwundoh F, Enver M, Lee A, Nargund V, Oliver T, Ben-Shlomo Y. Risk and presenting features of prostate cancer amongst African-Caribbean, South Asian and European men in North-east London. *BJU International *2006, **98**:1216–1220. [24]

The populations of Black men in the *United Kingdom *(UK) provide a very interesting population to study because they are mostly Afro-Caribbean and West African men. Thereby, they uniquely reflect a microcosm of the West African and Caribbean populations described above. Ethnic variations in prostate cancer have just recently been studied in the UK, with the landmark study of *The Prostate Cancer in Ethnic Subgroups *(PROCESS) group [24,25]. Chinegwundoh *et al *[24] published an earlier study in 2006. The researchers conducted a population-based audit of new prostate cancer diagnosis between January 1, 1999 and December 31, 2000 for the following regions: the London Boroughs of Tower Hamlets; Hackney; Newham; and the City of London. The total population in these regions was over 600,000 with an ethnic classification of White (British, Irish, "other" White), Black (Black Caribbean, Black African, Other Black, White and Black African mixed, White and Black Caribbean mixed), and Asian (Indian, Pakistani, Bangladeshi, other Asian, White & Asian mixed). The confirmed prostate cancer cases included 248 European men, 91 African-Caribbean men, and 20 South Asian men. These ethnic groups were compared on age-adjusted incidence rates for prostate cancer. The author's findings are summarized in Figures 1 and 2. African-Caribbean men had the highest age-specific incidence rates compared to European and Asian men. Interestingly, the relative risk for prostate cancer for the African-Caribbean men was three times that of European men. As noted by the authors, this risk is more than the risk documented for African American men in the US. A reason that has been suggested for the larger relative difference between African-Caribbean and European men is a lower prostate cancer rate for White men in the UK [25].

**Figure 3:**
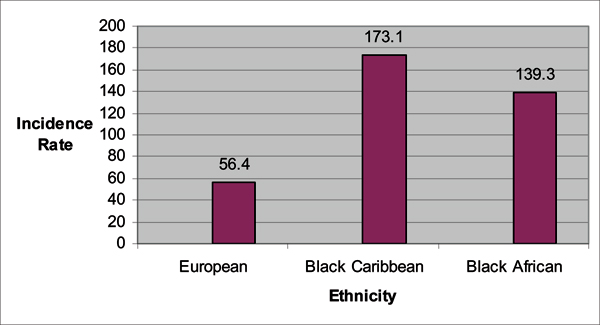
**Age-adjusted prostate cancer incidence rates comparing UK European, African Caribbean, and South Asian men, age 40 and above, between 1997 and 2001**. *Incidence rate is per 100,000. Source: Ben-Shlomo Y, Evans S, Ibrahim F, Patel B, Anson K, Chinegwundoh F et al: The Risk of Prostate Cancer amongst Black Men in the United Kingdom: the Process Cohort Study. *Eur Urol *2008, **53**:99–105. [25]

**Figure 4:**
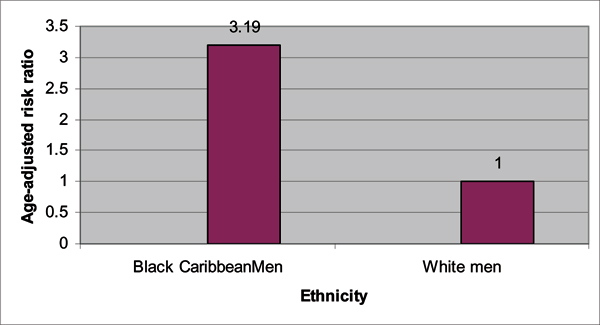
**Age-adjusted risk ratios for prostate cancer comparing UK Black Caribbean and White men, age 40 and above, between 1997 and 2001**. *P value < 0.0001. Source: Ben-Shlomo Y, Evans S, Ibrahim F, Patel B, Anson K, Chinegwundoh F et al: The Risk of Prostate Cancer amongst Black Men in the United Kingdom: the Process Cohort Study. *Eur Urol *2008, **53**:99–105. [25]

**Figure 5:**
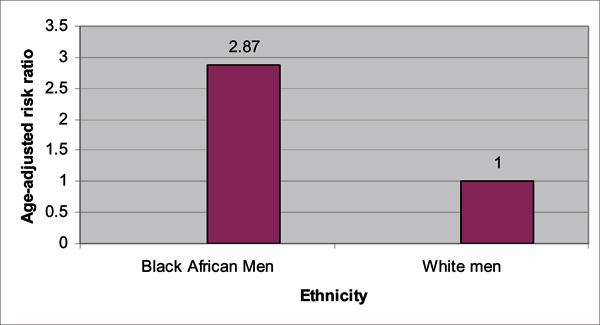
**Age-adjusted risk ratios for prostate cancer comparing UK Black African and White men, age 40 and above, between 1997 and 2001**. *P value < 0.0001. Source: Ben-Shlomo Y, Evans S, Ibrahim F, Patel B, Anson K, Chinegwundoh F et al: The Risk of Prostate Cancer amongst Black Men in the United Kingdom: the Process Cohort Study. *Eur Urol *2008, **53**:99–105. [25]

**Figure 6:**
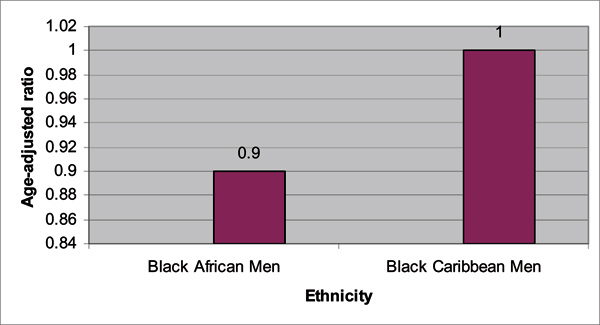
**Age-adjusted risk ratios for prostate cancer comparing UK Black African and Black Caribbean men, age 40 and above, between 1997 and 2001**. *P value = 0.35. Source: Ben-Shlomo Y, Evans S, Ibrahim F, Patel B, Anson K, Chinegwundoh F et al: The Risk of Prostate Cancer amongst Black Men in the United Kingdom: the Process Cohort Study. *Eur Urol *2008, **53**:99–105. [25]

In a follow-up study published this year, the PROCESS study group [25] conducted a retrospective population-based audit of prostate cancer incidences in North Bristol, South West London, South East London, and North East London from 1997 to 2001. Within this 5-year period, 2 140 prostate cancer cases were reported with: 61.4% White, 20% Black Caribbean, 4.8% Black African, 0.5% Black unclassified, 6% other ethnic groups, and 7% un-coded ethnicity. A summary of the findings are provided in Figures 3, 4, 5, 6. The authors confirmed their previous findings in the Chinegwundoh *et al.'s *study [24]. An interesting component of the current study is the comparison of the Black Caribbean group to the Black African group, with no statistically significant difference on age-adjusted prostate cancer rates between both groups.

## Discussion and conclusion

Based on the growing literature on the disproportionate burden of prostate cancer among other Black men of West African ancestry, it is clear that this clearly follows the path of the TAS. The primary West African source populations for the TAS were the African countries – Benin, Nigeria, Ghana, Gambia and Senegal. Our critical review of the literature on these West African countries found that the true prostate cancer rates reported for West Africans by the WHO [11] may be underestimated. This is not surprising since there are no data available on cancer incidence and mortality for most of the West African countries. Contrary to the global ranking, several published studies, including our previous review article for Nigeria [8], indicate higher incidence of prostate cancer in Nigeria [26-40] and Ghana [13,14]. There are no published literature for Benin, Gambia and Senegal. Prostate cancer morbidity and/or mortality data from the Caribbean Islands [10,15-23] and the UK [24,25] also provide similar or worse prostate cancer burden for Black men of West African ancestry.

Table 1 provides a summary of the numerical findings of published incidence and prevalence rates for prostate cancer in Black men of West African Ancestry. It is worth noting that the direct comparisons of these numerical findings may not be valid given the differences in the data reporting methods for the incidence and prevalence of, and mortality from prostate cancer in these countries. The validity of the data reporting methods are further limited in African countries due to underreporting [26], lack of appropriate diagnosis [26,27,37,41], limited access to care [41], differences in technical manpower and infrastructure [41], and the quality of cancer data systems [41-45].

The consistent higher incidence of prostate cancer relative to other groups, observed in populations of West African descent may be attributed to the fact that these populations share ancestral genetic factors that increase susceptibility to prostate cancer. However, the likely variability in risk across these populations of African men around the world may also suggest a potential and important influence of environmental/lifestyle factors acting on prostate cancer risk in these susceptible populations. To date the disparate incidence of prostate cancer in African American men in the US compared to their Caucasian counterparts is poorly understood. Although modifiable exposures related to lifestyle or environment is believed to play a major role in the etiology of prostate cancer, the specific causal factors remain elusive. To identify these causal factors and to better understand and address the global prostate cancer disparities seen in Black men, it is important to distinguish genetic and environmental determinants of prostate cancer in men of West African ancestry, especially the original TAS source population for African American men. As delineated by Odedina, Ogunbiyi and Ukoli [8], even with the available and emerging data, there are more questions than answers at this time: *Does the similar genetic characteristic of Black men of West African ancestry put them at higher risk for prostate cancer compared with other groups? Are there common environmental conditions/lifestyle factors among these men that may be responsible for the prostate cancer burden experienced by this group? What is the relative contribution of genetic, lifestyle and environmental factors in prostate cancer incidence and mortality among this group? *Our report establishes and provides a clear need, in addition to delineating the next steps, for a systematic and comprehensive evaluation of these populations that can facilitate a better understanding of the etiology of unequal burden of this disease in African American men.

## Competing interests

The authors declare that they have no competing interests.

## Authors' contributions

FTO, NK, MLF, and RRR conceptualized and designed the study. FTO was responsible for the background on the Transatlantic Slave Trade and African American men ancestry. NK and DY were responsible for establishing the inclusion and exclusion criteria for the study. FTO and NK reviewed the articles to ensure the articles meet the inclusion criteria. MLF, LBG, and BR conducted the review on the burden of prostate cancer in U.S. Black men. FTO and TOA reviewed the articles on the burden of prostate cancer in West African men. FC conducted the review on the burden of prostate cancer among the European ancestral groups of U.S. Black men. RR and RRR reviewed the articles on the burden of prostate cancer in the Caribbean ancestral groups of U.S. Black men. All the authors reviewed and approved the manuscript.
